# Development, optimization, and validation of novel anti-TEM1/CD248 affinity agent for optical imaging in cancer

**DOI:** 10.18632/oncotarget.2188

**Published:** 2014-07-08

**Authors:** Chunsheng Li, Junying Wang, Jia Hu, Yi Feng, Kosei Hasegawa, Xiaohui Peng, Xingmei Duan, Aizhi Zhao, John L. Mikitsh, Vladimir R. Muzykantov, Ann-Marie Chacko, Daniel A. Pryma, Steven M. Dunn, George Coukos

**Affiliations:** ^1^ Ovarian Cancer Research Center, University of Pennsylvania; ^2^ Department of Cancer Biology, University of Pennsylvania; ^3^ Institute for Translational Medicine and Therapeutics, University of Pennsylvania; ^4^ Nuclear Medicine & Clinical Molecular Imaging, Department of Radiology, University of Pennsylvania; ^5^ Department of Immunology, Norman Bethune College of Medicine Jilin University; ^6^ Saitama International Medical Center Saitama Medical University; ^7^ Ludwig Cancer Research Center, University of Lausanne

**Keywords:** scFv, TEM1, endosialin, optical imaging

## Abstract

Tumor Endothelial Marker-1 (TEM1/CD248) is a tumor vascular marker with high therapeutic and diagnostic potentials. Immuno-imaging with TEM1-specific antibodies can help to detect cancerous lesions, monitor tumor responses, and select patients that are most likely to benefit from TEM1-targeted therapies. In particular, near infrared(NIR) optical imaging with biomarker-specific antibodies can provide real-time, tomographic information without exposing the subjects to radioactivity. To maximize the theranostic potential of TEM1, we developed a panel of all human, multivalent Fc-fusion proteins based on a previously identified single chain antibody (scFv78) that recognizes both human and mouse TEM1. By characterizing avidity, stability, and pharmacokinectics, we identified one fusion protein, 78Fc, with desirable characteristics for immuno-imaging applications. The biodistribution of radiolabeled 78Fc showed that this antibody had minimal binding to normal organs, which have low expression of TEM1. Next, we developed a 78Fc-based tracer and tested its performance in different TEM1-expressing mouse models. The NIR imaging and tomography results suggest that the 78Fc-NIR tracer performs well in distinguishing mouse- or human-TEM1 expressing tumor grafts from normal organs and control grafts in vivo. From these results we conclude that further development and optimization of 78Fc as a TEM1-targeted imaging agent for use in clinical settings is warranted.

## INTRODUCTION

Antibody-based diagnostic and therapeutic strategies are entering center stage as cancer treatment options. While intact monoclonal immunoglobulins (mAbs) constitute the majority of US Food and Drug Administration (FDA) approved antibody-based therapeutics, minimal fragment derivatives, such as the single chain Fv (scFv) that target tumors have demonstrated promising therapeutic potential in recent clinical trials (reviewed here [1]). Rapid advances, both in the screening of human-derived antibody libraries, and in the engineering of scFv-based protein derivatives now provide great opportunities for developing highly specific multivalent antibody fragments tailored to meet the stringent functional demands required for various clinical applications.

Immuno-imaging with tumor biomarker-specific antibodies is a rapidly developing research area. Immuno-imaging can provide vital information on tumor stages and tumor response without invasive treatments, and it can also help to screen for patients that are likely to benefit from specific targeted therapies. In fact, several ^124^I- or ^89^Zr-labeled mAb radiopharmaceuticals have been approved by FDA in immuno-PET (positron emission tomography) and SPECT (single-photon emission computed tomography) imaging applications [2]. Recently, optical imaging has attracted increasing attention as an alternative imaging approach for cancer. Immuno-optical imaging requires no radioactivity, is able to acquire real-time intraoperative imaging information, and can be used for both two-dimensional imaging and three-dimensional tissue reconstruction [3]. For example, intraoperative detection of breast cancer [4] and lymph node mapping [5-9] within clinical settings is regularly performed using optical imaging with indocyanine green (ICG). Hence, due to its non-invasive and non-radioactive advantages, optical imaging using tumor biomarker-specific antibodies is particularly well suited to serve as a complimentary imaging technique to PET/SPECT. In particular, by exploiting the unique properties of light at near infrared (NIR) wavelengths, the “NIR optical biopsy”, in combination with endoscopy or camera, allows direct visualization of marker expression in live tissues *in vivo*, thus circumventing the need for histo- or cytological staining of specimens taken from target tissues. In addition, as optical imaging is less expensive and imposes no radioactive burden on the subject of study, it can be especially attractive as a tool for antibody development and optimization.

The success of immuno-optical imaging relies critically on the identification of robust biomarkers and the subsequent development of antibodies with the appropriate stability and pharmacokinetic profiles for imaging applications. In recent years, several markers associated with the tumor vasculature (TVM) have received considerable attention as therapeutic targets [10-15]. Key drivers have been their low rates of mutation relative to cancer cells [16, 17], direct accessibility to exogenous agents via the circulation, and their compelling association with angiogenic processes shown to be essential for tumor disease progression. Blockade of the latter is clearly correlated with tumor regression and is an established anti-cancer strategy [18, 19]. To this end, multiple lines of evidence have suggested that Tumor Endothelial Marker 1 (TEM1/endosialin/CD248) is a promising TVM target: TEM1 is implicated in neo-angiogenesis [20, 21], vascular cell adhesion and migration [22, 23], and tumor progression [24]; in breast cancer, TEM1 overexpression correlates with lymph node metastasis, recurrence and death [25]; in ovarian cancer, increased TEM1 expression was found in the endothelial cells and vasculature-associated leukocytes in the tumor microenvironment [26]; the expression levels of TEM1 are considerably higher in the tumor vasculatures of various cancers [13, 20, 21, 27-31] but below the detection limit [20, 21] in normal adult tissues; and TEM1 knockout did not impair any normal biological processes in mice [32]. In addition to its vascular expression in ovarian and other carcinomas, TEM1 has been shown to be upregulated in the tumor cells of sarcomas [33, 34]. Further, a humanized mouse monoclonal antibody targeting TEM1, MORAb-004 [21], is currently undergoing phase-1 clinical trials for the treatment of patients with various cancers. We previously reported the first immuno-PET imaging study in preclinical animal models using this mAb [35, 36]. However, despite the obvious therapeutic interest, suitable imaging tools to explore the potential of TEM1 as a diagnostic marker have to-date not been reported otherwise.

We have recently isolated a TEM1-specific fully-human scFv, scFv78, which was shown to bind with high affinity to both human and mouse TEM1 [37], and thus to have relevance as a test molecule in preclinical models. It is widely considered, however, that the functional efficacy of the scFv format is often limited due to its small size and monovalency (reviewed here [1]). We have sought to exploit the TEM1 recognition potential of scFv78 for immuno-imaging applications by fusing it to an immunoglobulin Fc domain. Fc-fusion proteins often demonstrate a number of beneficial biological and pharmacological characteristics: firstly, the plasma half-life of the Fc-containing fusion proteins are often significantly improved due to slower renal clearance and enhanced interaction with salvage Fc receptors [38]; secondly, the presence of an Fc domain allows easy purification of the fusion protein by protein-G/A affinity chromatography [39]; finally, the Fc fragment is dimeric in the native state which serves to increase the avidity of the scFv fusion partner, potentially improving potency (sensitivity) and performance in demanding applications, such as those encountered in the clinic. Currently, several Fc-fusion proteins have undergone clinical testing and have demonstrated favorable pharmacokinetics, half-life and avidity towards their antigens (reviewed here [40]).

In this study we have engineered a panel of TEM1-specific, fully-human, bivalent antibody-like reagents by fusing scFv78 with different domain components from the human immunoglobulin G1 (IgG1) Fc. We evaluated the affinities and stabilities of scFv78 and its derivatives and characterized their pharmacokinetic (PK) profiles in naïve and TEM1-positive graft bearing animals. Through these studies, we identified one construct, 78Fc, as being a strong candidate for immuno-imaging applications. In two preclinical models where mouse TEM1 is either spontaneously upregulated or human TEM1 is expressed in tumor vascular xenografts, we show that *in vivo* NIR optical imaging using fluorochrome-labeled 78Fc can distinguish high-TEM1 expressing tumor grafts from normal organs. These findings support further clinical evaluation of 78Fc as an optical imaging agent in cancer patients.

## RESULT

### Development and purification of oligomeric scFv78 -Fc fusion proteins

**Fig.1:**
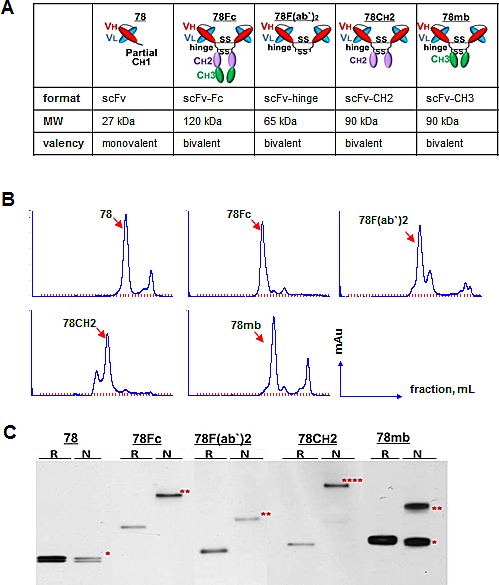
Development, purification, and characterization of scFc78 fusion proteins A, Schematic representation of the domain organization and the predicted molecular weights and valencies of scFv78 and the four scFv78 fusion derivatives. B, SEC analyses of affinity purified scFv78 and the four scFv78 fusion derivatives. C, multivalent status of scFv78 and the four scFv78 fusion derivatives by Western blot analysis with anti-HA antibody. R: reducing conditions, N: non-reducing conditions. * monomer, ** dimer, **** multimer.

Since the practical utility of many scFvs are often limited due to their small size, structural instability due to relatively weak variable domain interactions, and monovalency [1], we sought to construct novel oligomerised scFv78 variants more suitable for therapeutic and prognostic (theranostic) applications. To achieve this goal, we designed four multivalent scFv-Fc fusion proteins: 78F(ab`)_2_, 78CH2, scFv78-minibody (78mb), and scFv78-Fc (78Fc) (Fig 1A). While 78F(ab`)_2_ was generated by linking two scFv78 together via the IgG1 core hinge region (CPPCP), the other three variants were constructed by fusing different Fc regions to the C-terminal of scFv78. The calculated molecular weight of bivalent molecules of 78F(ab`)_2_, 78CH2, 78mb, and 78Fc are 65kDa, 90kDa, 90kDa, and 120kDa, respectively. A HA tag was added to the N terminus of the proteins for easy purification and detection, and upstream addition of the signal peptide from Ig KappaV enabled the fusion proteins to be secreted and easily purified from the media of the host 293T expression cells (sup Fig 1). Fusion proteins were purified by incubating the conditioned culture media with anti-HA affinity matrix beads. For all fusion proteins, we were able to purify 0.5-1mg/L protein at a purity >90%. Since the size exclusion HPLC (SE-HPLC) analysis of the purified proteins revealed additional peaks, suggesting the presence of aggregates/multimers of the proteins (Fig 1B), we further analyzed the quarternary status of the proteins by polyacrylamide gel electrophoresis. Under reducing conditions, the migration of all scFv derivatives appeared consistent with their calculated molecular weights (Fig 1C). Under non-reducing conditions, while scFv78 remained monovalent, we observed apparent oligomerisation of the fusion proteins: for 78Fc and 78F(ab`)_2_, the majority of protein appeared dimeric; for 78CH2, the majority (>90%) of protein migrated with an apparent mass consistent with a tetramer; and for 78mb, about 40% of the protein remained monomeric.

### scFc78-Fc fusion proteins have higher avidity to TEM1 than scFv78

**Fig.2:**
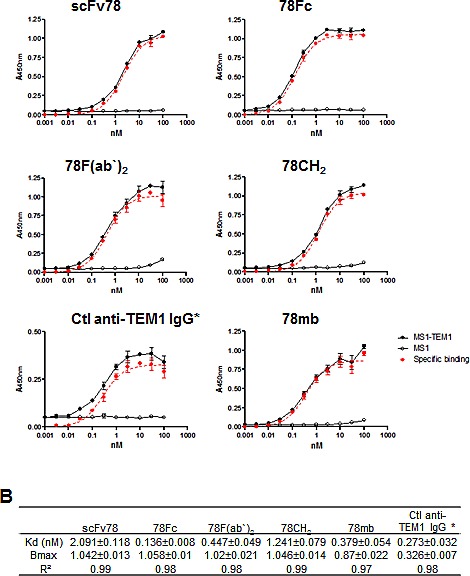
scFv78 fusion proteins demonstrate higher avidity to cell-surface TEM1 A, The dose-dependent binding curves of scFv78 and its derivatives. Cell-surface binding activity in control (MS1) or TEM1-expressing cells (MS1-TEM1) was measured by a live cell-based ELISA assay. TEM1-specific binding was calculated as the difference between binding to the control and TEM1-expressing protein. The control anti-TEM1 IgG is a biotinylated humanized mouse IgG1. B, A summary of the calculated K_d_ and B_max_ of the scFv78 and scFv78 derivatives. Anti-HA HRP was used as the secondary antibody in ELISA for all proteins except for the control anti-TEM1 IgG which required strepavidin-HRP. * indicates the different secondary antibody used.

It is well established that increases in valency can improve the avidity of an antibody. To measure the avidities of scFv78 and its derivatives under conditions that are more relevant to *in vivo* settings, we established a live-cell ELISA assay to measure the binding of the fusion proteins to cell-surface TEM1. Briefly, we first modified Mile-Sven1 (MS1), a TEM1-negative endothelial cell line, to express human TEM1 at a moderate level, with the saturated maximal binding capacity (B_max_) of ~4 × 10^5^ per cell. Different concentrations of scFv78 derivatives were then incubated with either control or TEM1 positive MS1 cells. Following washing, remaining molecules bound to the live cells at each concentration were detected by ELISA. Specific binding was observed when the concentration of fusion protein was as low as 0.1 nM, and non-specific binding was not observed below 10 nM (Fig 2A). While all samples tested have comparable B_max_, the fusion proteins all have lower apparent K_d_ values than scFv78, consistent with higher oligomeric avidities to TEM1 (Fig 2B). However, the apparent oligomerisation of 78CH2 does not translate into the expected avidity gain, suggesting that this species may have steric or structural issues. Among all antibodies tested, 78Fc demonstrated the lowest K_d_ value in sub-nanomolar range, which was ~15-fold lower than that of scFv78 (Fig 2B).

### The stability and pharmacokinetic profiles of scFv78 fusion proteins

**Fig.3:**
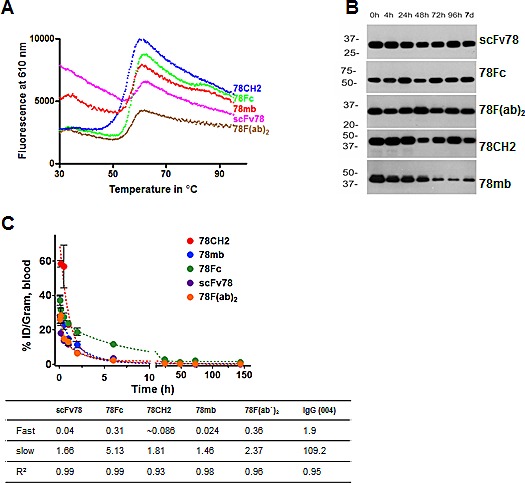
The stability and pharmacokinetic profiles of scFv78 and its derivatives A, Thermal stability curves of the antibody variants. Differential scanning fluorimetry was used to measure protein aggregation and degradation. B, Serum stability assay of scFv78 and its derivatives. Purified antibody variants were incubated with 100% human serum for up to 7 days at 37°C. Samples taken at different time points were then analyzed by Western blot with anti-HA antibody. C, Pharmacokinetics of ^125^I-labeled anti-TEM1 variants in naïve nu/nu mice. The half-lives (fast and slow, in hours) deduced from the study is underneath.

Good biophysical stability and appropriate serum half-life are generally considered important prerequisites for antibodies or antibody products destined for clinical applications. To evaluate the stability of the scFv78 fusion proteins, we first measured their thermal stability by incubating the purified proteins with SYBR orange under a temperature gradient from 20°C to 99°C. Following incubation, the samples were analyzed by differential scanning fluorimetry (DSF), an assay employed to estimate protein degradation/unfolding and aggregation. The thermostability curves revealed that the melting temperatures (T_m_) of scFv78 and its derivatives were comparable (Fig 3A).

Next, we tested the *in vitro* serum stability of scFv78 fusion proteins by incubating the proteins in 100% human serum at 37C for up to 7 days. Samples were collected at different time points during incubation, and HA-agarose beads were used to capture the fusion proteins. Serum stabilities were measured by quantifying the amount of bead-bound recovered protein by western blot analysis. In the presence of serum at 37^o^C, recoverable levels of scFv78, 78F(ab`)_2_, 78CH2, and 78Fc remained relatively consistent throughout the 7-day incubation; yet, the amount of bead-bound 78mb decreased significantly after two days of incubation (Fig 3B). This apparent instability of 78mb may be a consequence of its heterogeneous composition (Fig 1C). These observations suggest that, with the exception of 78mb, the scFv78 fusions possess sufficient physicochemical stability to make them attractive candidates for *in vivo* applications.

Finally, we conducted *in vivo* pharmacokinetic studies to determine the blood clearance rates of scFv78 fusion proteins. scFv78 and its fusion proteins were first radiolabeled with iodine-125 (^125^I). When injected into naïve nu/nu mice, serum concentrations of these ^125^I–labeled Fc fusion proteins decreased in a typical biphasic manner (Fig 3C). While scFv78 was rapidly eliminated from circulation, presumably via glomerular clearance, scFv78-derived fusion proteins were cleared from plasma much slower (fast-phase t_1/2_ of scFv78 and 78Fc are 0.04 h and 0.31 h, respectively). Importantly, the slow-phase half-life of 78Fc is about 5.1 h, long enough to serve as an imaging tracer but short enough to avoid antibody-retention-associated imaging background as seen with many full-length immunoglobulins.

### Codon-optimization, scale-up production and purification of the 78Fc fusion protein

**Fig.4:**
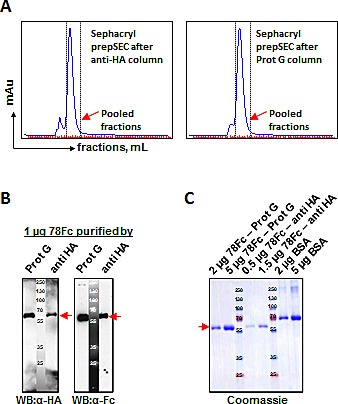
Comparison of two purification methods for scale-up production of 78Fc fusion protein after codon-optimization Scale up production of fusion protein 78Fc was performed after codon optimized by adapting codon bias of Homo sapiens genes with GeneOptimizer (Life technology). A, Elution profiles on SEC columns after affinity purification with anti-HA (left panel) or Protein G (right panel) methods. 78Fc protein elution fractions which were pooled (red arrowhead) are indicated with dotted-lines. B and C, Comparison of the purification efficiencies of anti-HA and Protein G methods by Western blot analysis (B) and Coomassie blue staining (C). A yield of 15-20 mg/L were achieved post optimization from 293 Freestyle cells (Invitrogen) compare with 0.5-1mg/L before optimization with purity >95%.

The observation that 78Fc displays encouraging *in vitro* thermal and serum stability behavior, in addition to possessing the highest avidity to TEM1 and a favorable murine PK profile, prompted us to investigate further the suitability of 78Fc for imaging applications by extending the study into preclinical mouse models. As a first step, we sought to improve the HEK cell expression yield and purification efficiency of 78Fc. We first improved the *Homo sapiens* Codon Adaptation Index (CAI) score of the 78Fc coding sequence to 0.96 (GeneOptimizer; Life Technologies). Subsequently, *cis*-acting sites (such as splice sites, RNA instability motifs, TATA box, GC-hi and AT-hi regions, ribosomal entry sites) that could potentially inhibit protein expression were removed wherever possible. Finally, we adjusted the GC content to 63% to prolong mRNA half-life. These measures were sufficient to increase the yield of 78Fc from 0.5-1 mg/L for the un-optimized gene, to 15-20 mg/L for the optimized gene expressed in HEK 293 Freestyle cells. We next sought to compare the purification capture efficiencies of anti-HA and Protein G matrices. 78Fc protein was first enriched with anti-HA agarose or Protein G, and the purified proteins were then subjected to size exclusion chromatography (SEC) prior to concentration of peak fractions (Fig 4A). The yields and purities of the final concentrated proteins were compared by SDS-PAGE using Western blot analysis and Coomassie dye staining (Fig 4B, C). The results indicated that both affinity approaches were equally effective for purification with final purities exceeding 95%. Since purification using Protein-G is more cost-effective, we decided to use Protein-G capture combined with SEC to purify large quantities of 78Fc for *in vivo* testing.

### 78Fc biodistribution *in vivo*

**Fig.5:**
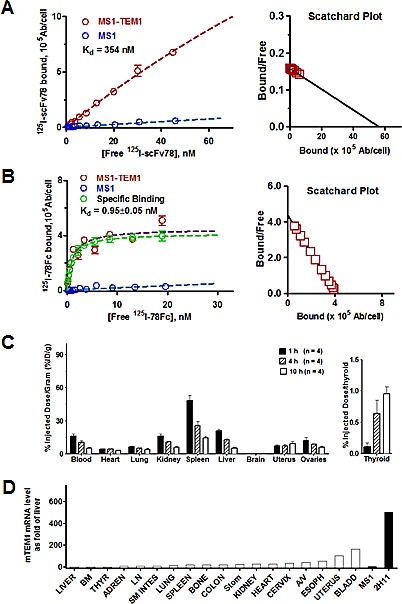
78Fc demonstrates little off-target binding in major mouse organs *in vivo* A, B K_d_/B_max_ binding studies of radiolabeled anti-TEM1 scFv78(A) and 78Fc(B). The avidity of 78Fc to TEM1 was not significantly affected by ^125^I labeling. C, Biodistribution profile of ^125^I-78Fc protein in naïve mice, showing no TEM1-specific accumulation in major organs. Left, % injected dose/gram in major organs; right, % injected dose/gram in thyroid. D, muTEM1 mRNA level in normal mouse organs determined by qPCR. TEM1 expression in liver was set as 1 (open bars). MS1 and 2H11 (dark bars) are used as negative and positive controls.

Since scFv78 binds to both human and mouse TEM1 [37], we reasoned that mouse would be a valid preclinical model species for evaluating the tissue uptake and biodistribution of 78Fc. To characterize the tissue uptake and biodistribution of 78Fc in naïve mice, we first radiolabeled 78Fc and scFv78 with ^125^I. While the affinity of scFv78 to TEM1 decreased significantly upon conjugation (Fig 5A), the apparent affinity of radiolabeled 78Fc was still in the nanomolar range, only slightly lower than that of its unlabeled counterpart (Fig 5B). The radiolabeled 78Fc was injected into naive mice and samples from multiple organs, including full blood, thyroid gland, heart, lung, kidney, spleen, liver, brain, uterus and ovary, were collected at different time points. The quantity of 78Fc protein present in the various tissues was assumed to correlate with the measured radioactivity. While there was an acute increase of measurable radioactivity following the 78Fc injection, the signals observed in normal mouse organs were seen to decrease rapidly with time, consistent with the absence of target-mediated retention and reflecting the low detectable levels of TEM1 mRNA in these tissues (Fig 5D). Interestingly, one exception was the uterus where apparent retention/accumulation of label appeared to correlate with a modest level of mouse TEM1 mRNA. It is worth noting that while kidney and liver are considered major clearance routes for scFv-Fcs, we did not detect any signal enrichment in these two organs. In tumor-bearing animals, we observed that 78Fc was not retained in normal tissues but was clearly enriched in TEM1-expressing tumors (Sup Fig 2). The observation that low levels of tracer accumulation occurred in the thyroid (Fig 5C), a phenomenon which we have observed previously [38, 39], can be attributed to the dehalogenation of the ^125^I –labeled 78Fc. Taken together, this data suggests that off-target or non-specific binding of 78Fc is unlikely to be a confounding factor for *in vivo* imaging.

### 78Fc as a tracer for optical imaging in murine TEM1-expressing lung tumor models

**Fig.6:**
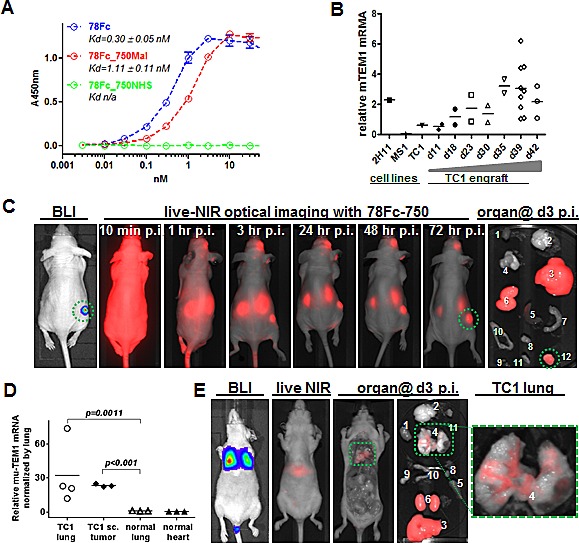
78Fc as an NIR optical imaging tracer in a murine TEM1-expressing lung cancer xenograft model A, Live cell ELISA analysis of 78Fc labeled with Vivotag750 via either maleimide (red) or NHS (green) linkage. Blue indicates the binding activity of unlabeled 78Fc. B, TC1-derived subcutaneous tumor grafts express high level of muTEM1. 10^4^ TC1 cells were injected subcutaneously into B6 mice and tumors were harvested at the indicated time (up to 6 wks). muTEM1 mRNA levels in tumor and parental cells were measured by qRT-PCR, and 2H11 and MS1 cells were used as positive and negative controls, respectively. C, in vivo bioluminescence imaging (BLI) and 78Fc750-based live NIR optical imaging in a TC1 subcutaneous tumor model (n=5). 78Fc750 was given via retro orbital injection (5 mg/kg in 100 uL) and a longitudinal study of NIR imaging was performed up to 3 days post injection (n=5). Green circle, muTEM1+ positive tumor. 1. heart; 2.brain; 3. liver; 4. lung; 5. spleen; 6. kidney; 7. small intestine; 8. bladder; 9. ovary; 10. uterus; 11. thyroid; 12. TC1 sc. xenograft. Residual tracer was observed at the injection site (retro-orbital complexes). D, TC1 lung metastatic lesions express higher levels of muTEM1 than normal tissue in the lung. E, in vivo bioluminescence imaging (BLI) and 78Fc750-based live NIR optical imaging in a TC1 lung metastasis model. 78Fc750 was given via retro orbital injection (5 mg/kg in 100 uL) and a longitudinal study of NIR imaging was performed at 3 days post injection (n=5). From left: ventral view of mouse BLI showing TC1 lung metastasis at day0; live animal NIR at day2 p.i. of 78Fc750; ventral view at day3 p.i. (postmortem) of same animal with liver and kidneys removed to show lung signal; all harvested organs including liver and kidneys; close up view of the lung. Green box, muTEM1+ positive lung tumor. 1. heart; 2.brain; 3. liver; 4. lung; 5. spleen; 6. kidney; 7. small intestine; 8. bladder; 9. ovary; 10. uterus.

As a rapidly developing technology, optical imaging is becoming a promising diagnostic modality in cancer management. To evaluate 78Fc as an optical imaging tool, we first generated 78Fc-fluorochrome conjugates via two different coupling methods: N-hydroxysuccinimide (NHS)-esterification, and thiol (-SH, sulfhydryl) reactive maleimide chemistry. We chose VivoTag-S750 as the conjugating fluorochrome because the near infrared wavelength is ideal for both *in vitro* and *in vivo* biological imaging applications. In addition, with the fluorescence tomography (FMT) imaging systems, VivoTag-S750 can also provide quantitative, three-dimensional information of *in vivo* target tissues. While the fluorochrome-78Fc conjugate prepared with NHS chemistry effectively ablated the ability of 78Fc to bind to TEM1 (Fig 6A), the impact of maleimide coupling was far less dramatic, resulting in only a 3-fold reduction in apparent affinity for this conjugate (hereafter designated as 78Fc750) as compared to the underivatised 78Fc.

Next, we tested 78Fc750 in a TC1 mouse lung xenograft tumor model. While TC1 is a mouse lung metastasis cell line that expresses very little TEM1, TC1 tumor engrafts have high TEM1 mRNA expression (Fig 6B). To establish the TC1 tumor model for optical imaging, we stably transfected parental TC1 cells with firefly luciferase and sorted the luciferase-positive population. The luciferase expression allowed us to track tumor location and growth via bioluminescent signal detection in live animal imaging. Luciferase-positive TC1 cells (1 × 10^5^) were inoculated subcutaneously at a single site on the flanks of nude mice (n=5). One week after the inoculation, successful engraftment of TC1 tumors was confirmed by bioluminescent imaging and the animals were then injected with 78Fc750 intravenously into the retro-orbital complexes. Near infrared optical images were acquired from 10 minutes up to 3 days post-injection of the tracer. Encouragingly, these TC1 tumors containing elevated levels of TEM1 mRNA were easily distinguished from surrounding normal tissues by NIR optical imaging using the 78Fc750 conjugate (Fig 6C). However, it should be noted that significant NIR signals were observed in the liver and kidneys of these tumor-bearing animals (Sup Fig 3). Since we did not find any enrichment of the ^125^I-labeled 78Fc in the liver or kidney from the preceding biodistribution study, we attribute this apparent staining to the known instability of plasma accessible maleimide linkages [41] and to probable maleimide exchange reactions between78Fc750 and plasma components that direct the accumulation of the liberated Vivotag-S750 to the key sites of excretion and metabolism. Similar phenomena have been described elsewhere [42, 43].

In order to confirm the above findings we established an alternative TC1 lung metastasis mouse model by inoculating the luciferase-positive TC1 cells intravenously into nude mice. The TC1-derived metastatic lesions formed seven days after cell inoculation and were confirmed by bioluminescent imaging. The TEM1 mRNA expression in TC1 lung metastasis lesions was higher than in the surrounding lung tissue (Fig 6D). In this model, NIR optical imaging with 78Fc750 was able to clearly distinguish the TC1-derived lung lesions from surrounding healthy tissues (Fig 6E).

### 78Fc as a tracer for optical imaging in human TEM1-expressing tumor vascular model

**Fig.7:**
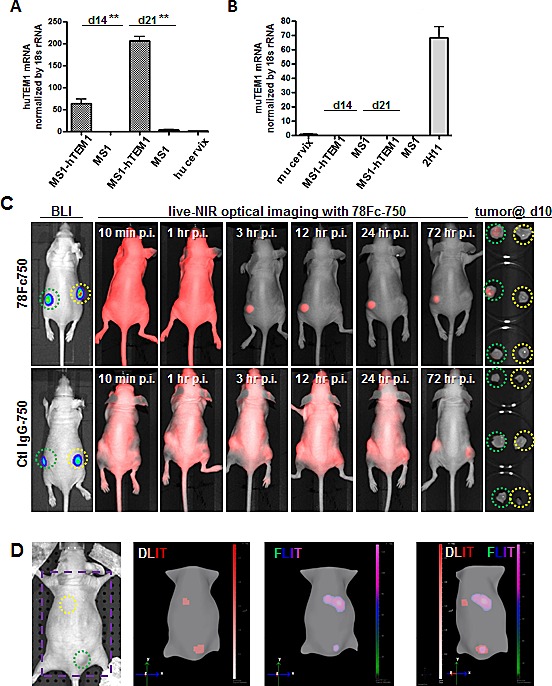
78Fc as an NIR optical imaging tracer in the human TEM1-expressing tumor vascular model Human (A) and mouse TEM1 mRNA (B) expression in control and hTEM1 expressing tumor grafts. TEM1 mRNA levels were evaluated with qRT-PCR. TEM1 expression in normal human or murine cervix was normalized to 1. 10^7^ MS1-huTEM1 or control MS1 cells were injected subcutaneously into nude mice on the left or right flanks, respectively (n=3), and tumor grafts were harvested after the indicated time (2-3 weeks). huTEM1-positive tumor grafts express muTEM1 at similar levels to that of control tumor grafts. C, in vivo bioluminescence imaging (BLI) and 78Fc750- or IgG-750-based live NIR optical imaging in huTEM1-positive and control tumor grafts (n=3 per group). Green circle, MS1-TEM1 tumor; yellow circle, MS1 control tumor. D (Also in Sup videos), 78Fc750 BLI and NIR tomographic imaging in mice grafted with MS1-huTEM1 (lower) and MS1 (upper) in nude mice. Bioluminescent signals overlap with infrared signals only in the huTEM1-positive tumors. From left, picture (area inside the purple square is used for 3D dual modality imaging to regenerate mice surface), 3D reconstructions of bioluminescence (DLIT) and NIR fluorescence (FLIT); superimposed DLIT with FLIT. n=5.

Next, we sought to evaluate the utility of 78Fc750 for imaging the human TEM1-expressing tumor vasculature using a xenograft model we established previously [35, 36]. In this model, luciferase-expressing control MS1 cells (MS1) or human TEM1-positive MS1 cells (MS1-hTEM1) were injected into opposite flanks of the same nude mice to grow control or TEM1-expressing tumor vascular grafts. Since mouse TEM1 expression remains low in these huTEM1-expressing tumor grafts (Fig 7A, B), we reasoned that this model could be used to evaluate the performance of 78Fc750 in detecting hTEM1-expressing grafts with NIR optical imaging. One week after grafting, the control and hTEM1-expressing MS1 vascular grafts were first assessed by bioluminescent imaging (BLI) (Fig 7C), before injecting 78Fc750 tracer for longitudinal NIR optical imaging. In common with the above biodistribution study in naive mice, the infrared signal in normal healthy organs, such as the heart, lung, or hTEM1-negative graft, was quickly cleared and became negligible within one day after tracer injection. In contrast, we detected intense and long-lasting fluorescence in hTEM1-positive grafts (Fig 7C).

Beyond 2-dimensional (2D) planar BLI, recent advancements include the ability to acquire 3-dimensional (3D) diffuse luminescence tomographic images (3D DLIT). To further confirm the specific localization of 78Fc to hTEM1-positive grafts, we conducted three-dimensional imaging with DLIT (bioluminescent source reconstruction for spatial information of TEM1+ and control engrafts) and FLIT (NIR fluorescence source reconstruction for spatial information of 78Fc750 tracer), and co-registration of the DLIT and FLIT voxels. We found that the bioluminescent and NIR fluorescence signals overlapped only at the site of hTEM1-positive grafts (Fig 7D, supplementary videos). Similar to the TC1 models, fluorescence was observed in the livers of these mice (Sup Fig 4). These observations demonstrated that 78Fc750 was specifically enriched in hTEM1-positive tissues.

## DISCUSSION

Antibodies now represent an established class of therapeutic reagents capable of inhibiting tumor growth and inflammatory disease progression in the clinic, with new registrations growing year on year. Increasingly however, antibodies are also being recruited as immune-modulating agents, vehicles for targeted delivery, and as imaging agents for detecting and staging tumors, and for monitoring treatment response. In recent years, indium-111 satumomab pendetide (CYT-103), technetium-99m nofetumomab merpentan (Verluma), technetium-99m arcitumomab (IMMU-4), indium-111 pentetreotide, and indium-111 capromab pendetide have been approved by the FDA as imaging agents for single-photon emission computed tomography (SPECT) in various cancers [2]. In addition, the radiolabelled derivatives of a growing number of therapeutic mAbs, including anti-CD44v6 (U36) [44], anti-cMet (DN30) [45], anti-carbonic anhydrase IX (G250) [46-48], anti-fibronectin (L19-SIP)[49], anti-IGF-1R (R1507) [50], anti-PSMA (J591 and 7E11) [51, 52], anti-CD105 (TCR105) [53], anti-EGFR (cetuximab) [54, 55], anti-CD20 (inbritumumab tiuxetan, rituximab)[56, 57], anti-VEGF (bevacizumab) [58, 59], and anti-HER2 (trastuzumab) [60-62] have been intensively evaluated in preclinical and clinical immuno-PET studies.

Although immuno-imaging with mAbs has met with some success [36], the large size of intact immunoglobulins effectively restricts their renal clearance which, together with Fc-recycling mechanisms, often results in prolonged circulating half-lives and a background of radioactivity that can persist for time frames that are incompatible with short duration high contrast imaging of specific target tissues. To reduce the half-life of circulating antibodies, extensive efforts have been directed towards developing antibody-based reagents with fewer domains and reduced size [63, 64]. The scFv minimal binding unit has featured heavily as a building block in such strategies (reviewed in [65]). Whereas scFvs themselves are considered poor reagents for imaging purposes, due mainly to a combination of rapid clearance and limited monovalent affinity, significantly better performance has been reported for several derivative scFv formats including scFv monomers conjugated to PEG or albumin, scFv dimers (diabodies), scFv-CH3 dimers (minibodies) and scFv-Fc dimers. One established strategy for domain reduction, and the subject of this work, is the design and construction of Fc-fusion proteins that comprise the Fc domain of an immunoglobulin and a (poly)peptide that possesses high affinity for the antigen of interest [66]. Such artificial constructs generally have reduced size and physicochemical and/or metabolic stability when compared alongside a native soluble immunoglobulin. Accelerated clearance for such molecules, although not expected per se, may arise from a combination of moderately improved renal clearance and/or degradative mechanisms, both endosomal and plasma-driven, which are dependent on the nature of the individual fusion. Fc-fusion proteins with such properties may display higher blood clearance rates and have improved utility as imaging reagents. Though TEM1 is a well-validated tumor vascular marker and a promising theranostic target, currently there is only one chimeric, hTEM1-specific mAb undergoing clinical trials in patients with multiple types of cancer. To maximize the translational potential of TEM1, we have identified a potent TEM1-specific scFv, scFv78, arising from yeast display and FACS-based screening [37], and have explored the contribution of four different valency-enhancing IgG1 Fc domain fusion arrangements to stability, avidity and pharmacokinetics.

For an antibody to perform well in imaging applications, it has to fulfill many criteria. Principally, it needs to maintain its avidity to the antigen after labeling. Here we observed that, unlike scFv78, which totally lost its binding to TEM1 upon radiolabeling, 78Fc retained high affinity binding following both radio- and fluorochrome- labeling. Additionally, an antibody needs to have a favorable PK clearance profile for imaging applications. Although murine PK data of antibody-related molecules is considered unreliable for extrapolating clearance rates to humans [67], the blood clearance rate of around 5 hours for 78Fc in mice observed in our study, suggests that it may be possible for patients to be imaged within 24 hours following the tracer injection. Finally, to minimize potential off-target toxicity and masking, an antibody reagent must show minimal retention in non-target tissues. The evaluation of this criterion is challenging in commonly used preclinical models as many antibodies recognize only their cognate human antigens. In our case, since scFv78 recognizes both human and mouse TEM1, we reasoned that a mouse model would be relevant and informative in evaluating the bio-distribution and imaging performance of 78Fc. In biodistribution studies, ^125^I-78Fc demonstrated negligible uptake by critical tissues such as heart, lung, kidney, liver, and brain, and this was also the case for NIR imaging with 78Fc750. The observed non-specific signals in liver and kidney were most likely due to liberation and metabolism of the tracer, which could potentially be addressed using more optimal conjugation and linker chemistries. This finding is consistent with the fact that most adult normal tissues express very low level of TEM1 [68], and it also suggested that 78Fc has very little off-target binding that can interfere with its imaging applications.

To further evaluate 78Fc as an imaging tool, we tested in two different mouse models: TC1 lung metastasis model, in which TC1 tumor grafts have high expression of mouse TEM1; and hTEM1 expressing tumor vascular model, in which human TEM1-expressing endothelia cells formed hTEM1 expressing tumor vasculature. In both models, there was only negligible amount of signals in normal mouse organs like heart, lung and brain; however, in TEM1-positive grafts, we observed strong and long-lasting near infrared signals. Therefore, it is strongly suggested that one of the scFv-78 based fusion protein we designed here may have a great potential as an optical imaging tool for clinical uses. One should note that unlike the biodistribution study with ^125^I-labeled 78Fc, where no normal organ showed any sign of antibody retention, our optical imaging studies revealed strong fluorescent signals in liver and kidney. This signal most likely reflects the known instability of plasma accessible maleimide linkages and maleimide exchange between 78Fc750 and plasma components, thusleading to the accumulation of the liberated fluorochrome at key sites of excretion and metabolism. In a previous study with a HER2/Neu antibody, maleimide-conjugated linkers were shown to readily exchange with reactive thiols on albumin, free cysteine and glutathione, with the local charge context of the cysteine conjugation site also influencing the stability of the succinimide ring [41]. Similar observations of apparent liver and kidney localization have been reported for other optical imaging tracer conjugates, for example the commercial product Transferrin-Vivo750 (Perkin Elmer) and related conjugates [42, 43]. Thus, the chemical and structural dynamics of the conjugation site can influence antibody conjugate performance by modulating the stability of the antibody-linker bond, and contributing to non-specific background fluorescence in whole-body imaging applications. Further studies using conjugates stabilized by alternative chemistries and fluorochromes with improved free clearance profiles are clearly warranted..

NIR imaging has generally been considered practical only for tumors close to the body surface due to signal penetration limitations. However, recent technological advances are now challenging this perception by extending the utility of the approach to the imaging of internal organs in both pre-clinical and clinical settings [69-74]. As of June 2014, NIR imaging features in 14 clinical trials in intraoperative or endoscopic settings (www.clinicaltrials.gov). Two trials, NCT02129933 and NCT02113202, are concerned with evaluating, respectively, Bevacizumab-conjugated NIR tracers in patients with esophageal cancer and adenomatous polyposis.. Based on the encouraging results we have obtained using 78Fc-based NIR imaging reagents in pre-clinical models, further development activities are underway in order to maximize the translational potential of this antibody.

In summary, optical imaging is emerging as an important alternative to established radio-imaging technology. Immuno-optical imaging can provide accurate and specific information regarding the target tissue without invasive treatment or exposing the patient to radioactivity. It can also provide real time, spatial information regarding biomarker expression, which can help physicians to make clinical decisions during operations and guide treatment. In the current study we have demonstrated encouraging preliminary optical imaging results for a novel anti-TEM1 Fc-fusion reagent in a preclinical setting. Further optimization of the fluorochrome and conjugation chemistry is clearly warranted. Additionally, given that 78Fc can distinguish both human and murine TEM1-positive lesions from control lesions and surrounding normal tissues, our study also paves the way for developing 78Fc as a tracer for TEM1-targeted immuno-PET in both preclinical and clinical applications.

## METHODS

### Reagents and antibodies

Goat pAb to HA tag (HRP) antibody was from Abcam. Streptavidin HRP was from BD Biosciences. Biotinylated MORAb004 (humanized mouse IgG1 recognize human but not mouse TEM1) was produced by Morphotek Inc. pcDNA 3.3/3.4 vector, FreeStyle™ 293 Expression medium, Freestyle MAX reagent and OPTI PRO serum free medium were from Invitrogen. Anti-HA affinity matrix and HA peptide were from Roche. Silverquest staining kit was from Invitrogen. Sureblue TMB microwell peroxidase substrate and TMB stop solution were from KPL. HA Tag IP/Co-IP Kit was from Pierce Thermo Scientific. VivoTag-S 750-MAL and VivoTag-S 750 fluorochrome is from Perkin Elmer. Taqman qRT-PCR primers are from Applied Biosystems.

Bicistronic lentiviral vector plasmid pKH-GFP-pUbi-EmeGFP for fusion protein expression was based on pHR-SIN backbone [75]. The Fc region used in the constructs is derived from locus AJ294730. The scFv78 or Fc-fusion cDNA was cloned into sites of lentiviral vector plasmid as MluI-NotI PCR fragments to replace the GFP fragment and to generate pKH-scFv78, 78Fc, 78F(ab)_2_, 78CH2, and 78mb respectively. The corresponding lentivirus was generated by calcium phosphate-mediated transient transfection of the antibody constructs together with the plasmids encoding the vesicular stomatitis virus G envelope *gag-pol* genes and pHRSIN-TEM1 into HEK-293T cells. Conditioned media containing virus were harvested 24 and 48 hours post-transfection, filtered with 0.45 μm filters, and frozen at -80 °C for further use.

### Cell lines

MILE SVEN 1 (ATCC) mouse endothelial cell line engineered to express DsRed and firefly luciferase, denoted MS1, has been described previously [35, 36]. MS1 was used for subsequent generation of the MS1-TEM1 cell line that expresses human TEM1 (hTEM1) and EmGFP, in addition to DsRed and fLuc [35, 36]. 2H11 murine endothelial cell line was used as muTEM1-positive control (ATCC). TC1, a C57BL/6 mouse lung adenocarcinoma cell line transformed with HPV-16 E6 and E7, was a generous gift from Dr Yvonne Patterson (University of Pennsylvania). The TC1 cell line engineered to express firefly luciferase fLuc, denoted TC1, was used in this study. MS1, MS1-TEM1, 2H11 and TC1 cells were cultured in RPMI1640 medium (Corning Cellgro, USA) containing 10% fetal bovine serum (FBS), 100 I.U/mL penicillin, and 100 μg/mL streptomycin. Cells were incubated at 37°C in a humidified atmosphere of 5 % CO_2_. 293-F cells were cultured in Gibco® FreeStyle™ 293 Expression Medium (Invitrogen) in a shaker flask and incubated in a 37°C incubator containing a humidified atmosphere of 8% CO_2_ in air on an orbital shaker platform rotating at 125rpm.

### Transfection and protein purification

Transfection of FreeStyle™ 293 cells was done according to the manufacturer's instruction. The culture supernatants were harvested at 72h post transfection and filtered through 0.45μm filter unit before purification. The supernatants were concentrated with 10k MWCO centrifugal filters (Millipore) and dialyzed with PBS before further purification. Small-scale protein purification was performed according to the manufacturer's protocol for anti-HA affinity matrix (Roche). Briefly, samples were incubated with Anti-HA affinity matrix and following three washes, specifically-bound proteins were eluted by HA peptide (Roche) in elution buffer and then dialyzed with PBS three times. The purity of each protein was analyzed using coomassie blue staining, Silverquest staining (Invitrogen) and SEC analysis.

### Codon optimization and scale up production and purification

The codon usage was analyzed with GeneOptimizer® (Life Technologies) and adapted to the codon bias of *Homo sapiens* genes. In addition, regions of very high (>80%) or very low (<30%) GC content were avoided where possible. During the optimization process, the following *cis*-acting sequence motifs were avoided: internal TATA-boxes, chi-sites and ribosomal entry sites; AT-rich or GC-rich sequence stretches; RNA instability motifs; repeat sequences and RNA secondary structures; splice donor and acceptor sites in higher eukaryotes. Post optimization, GC content was adjusted to prolong mRNA half-life [76]. Codon usage was adapted to the bias of *Homo sapiens* resulting in a CAI (codon adaptation index) value of 0.96.

Scale up protein production was done using the codon-optimized sequence in the pcDNA3.3 vector according to the manufacturer's protocols (Invitrogen). In brief, 6 days post-transfection, the supernatant was concentrated by tangential flow filtration before being applied onto anti-HA agarose or HiTrap Protein G HP for affinity enrichment. The binding buffer was 20 mM sodium phosphate and the elution buffer was 0.1 M Glycine (pH 2.4). All collection tubes contained 20% 1 M Tris-HCl (pH8.0) to neutralize the eluate fractions. Enriched protein was further purified by SEC on a Sephacryl S200 column pre-equilibrated with PBS (pH 7.5). Fractions containing the purified protein were pooled and dialyzed for 16 h against PBS (pH 7.5) before adjustment to 1mg/mL. Aliquots were stored at -80°C. Purity was determined as described above. Yield (mg/Liter culture) was quantified by spectrophotometry with a Nanodrop 2000 instrument (Thermo Scientific) and coomassie staining alongside a known loading of BSA.

### Western blot analysis and multivalent status assay of the antibody variants

Proteins were separated according to their molecular weight on 4-15% Mini-protean TGX precast gels (Bio-Rad) and transferred onto Immobilon-P transfer membrane (Millipore). Membranes were blocked for 1 h in 5% dried milk in TBST at room temperature and probed overnight at 4°C with rotation in primary antibody anti-HA-HRP (Abcam, 1:5000 dilute). After 3 washes, the bands were visualized using a chemiluminescent substrate (GE Healthcare).

### SEC analysis of purified fusion proteins

Recombinant antibody reagents including 78Fc, 78F(ab`)2, 78CH2, 78mb, and scFv78 were analyzed via a Superdex 200 10/300 gel-filtration column (GE Healthcare). 10-25μg of antibody was diluted with PBS to a final volume of 250 μL. The samples were spanned at 20,000 g for 5min before injection. The sample was applied via a 500 μL loop at a flow rate of 0.5 mL/min using an AKTA FPLC system (GE Healthcare). The eluting protein was detected by UV absorbance at 280 nm and the elution volumes were recorded for analysis. Column calibration with appropriate MW standards (Bio-Rad) was performed prior to and following each run in order to allow the estimation of antibody reagent MWs. Peak fractions were collected using the AKTA FPLC Frac-950 collector for further analysis.

### Radiolabeling and Immunoreactivity assays

Purified fusion proteins (50 μg) were radio-iodinated with ~18.5 MBq (0.5 mCi) of ^125^I-NaI (Perkin Elmer) in 0.01 N NaOH for 5 min using pre-coated iodination tubes (Thermo Scientific) as previously described [35]. Radiolabeled antibody was purified over a 2 mL desalting column (Thermo Scientific). Following trichloroacetic acid precipitation, radiolabeling efficiency was determined by radio-TLC.

Immunoreactivity was assayed as previously described [35]. Briefly, radio-iodinated proteins (5 nM − 0.016 nM) were incubated with an excess of MS1-TEM1 cells for 1 h. The mixture was centrifuged, and the activity remaining in the supernatant was counted. A more sensitive assessment of specific binding was done by live-cell radioimmunoassay (RIA). Cells were grown to confluence on 1% gelatin-coated 96-strip well microplates (Corning Life Sciences). For the binding assay, monolayers of cells were incubated with increasing concentrations of ^125^I-labeled proteins in assay buffer (5% FBS in RPMI 1040) in quadruplicate at 4 °C for 4 h. At the end of incubation, cells were washed five times with ice-cold wash buffer (3% BSA/PBS). The cell-associated radioactivity was measured by a gamma (γ) counter (Wizard 2470, Perkin Elmer) and was normalized to the total number of cells, as counted by a hemocytometer. Non-specific binding (NSB) was calculated by subtracting the radiolabel bound to control MS1 cells from the total binding. The data from the live-cell RIA experiments were analyzed by Scatchard analysis using Prism 5.0 (GraphPad) software to determine equilibrium binding constants and the number of functional binding sites. The apparent binding affinity, K_d_, for specific binding was calculated using non-linear regression analysis of a one-site binding hyperbola equation. B_max_ is the maximum number of binding sites per cell at the asymptotic maximum; K_d_ and B_max_ values are presented as the mean ± SD of three or more independent experiments, and each independent experiment was performed in quadruplicate.

### Blood pharmacokinetics (PK) and biodistribution studies

Naïve mice were administered ^125^I-labeled protein (0.185 MBq [5 μCi], 5 μg of protein, in 150 μL of saline) via the tail vein (0 h). Blood samples were collected at designated time points by retro-orbital bleed for assessment of blood activity levels. Blood activity levels were fitted to bi-exponential decay curves, and decay constants were calculated. Animals (n = 3–6 per group) were euthanized at 1, 2, 4, 24, 48, 72, and 144 h after injection. Organs and tumors were harvested and weighed, and the accumulated radioactivity was counted in a gamma counter.

### Live-cell ELISA-based direct binding assay

96-well plates were coated with 50 μL 2% gelatin (Sigma) at 37°C for 30 min. After discarding the gelatin, plates were washed with 200 μL PBS. MS1 and MS1-TEM1 cells were seeded in plates (2×10^4^ cells per well) and grown at 37°C overnight. Cells were incubated with 11 doses (starting from 100 nM, 3 × serial dilution) of antibody reagents in cell culture media at 4°C for 2 h. Plates were washed 3 times with 200 μL PBS and incubated with secondary antibody in cell culture media at 4°C for 1 h. For scFv78 fusion proteins, the secondary antibody was HA-HRP (Abcam, 1:1500 dilutions), for Biotinylated MORAb004, the secondary antibody was SA-HRP (Abcam, 1:1000 dilutions). Plates were washed with 200 μL PBS three times and then developed with 100 μL Sureblue TMB microwell peroxidase substrate (KPL) for 10 min. 100 μl TMB stop solution (KPL) was added to each well and the A450 was measured in a plate reader (BioTek EL800). ELISA binding curves were analyzed using Prism5.0 (GraphPad) software to determine relative binding affinity constants (IC_50_). Data were fitted using the “non-linear four-parameter logistic sigmoidal dose-response model”. The K_d_ value was then calculated from the IC_50_ value according to Cheng-Prusoff method [77]. K_d_ values are reported as the mean ± standard deviation (SD) of three independent experiments, and each experiment was performed in triplicate.

### Serum stability *in vitro*

500 ng of purified proteins (78Fc, scFv78, 78CH2, 78mb, or 78F(ab`)_2_) were added to 250 μL of 100% human serum and incubated at 37°C with shaking. An aliquot (25 μL, 100ng protein) of the mixture was taken out at each time point (4h, 24h, 48h, 72h, 96h, 7d) and diluted to 800 μL with PBS, and the HA-tagged protein was captured using the HA Tag IP/Co-IP Kit (Pierce). Briefly, each sample was loaded onto the column followed by the addition of 20 μL of anti-HA agarose. Columns were incubated with gentle end-over-end mixing for 2 h at 4°C, then washed 3 times with 0.5 mL PBST and added to 25 μL of 2× Non-Reducing Sample Buffer. The spin column/tube assembly was heated at 100°C on a heating block for 5 minutes and centrifuged for 10 seconds. The collected samples were electrophoresed in a 4-15% Mini-protean TGX precast gel (Bio-Rad) followed by blotting and western analysis.

### Thermostability assay

Thermofluor assays were performed to assess the thermal stabilities of purified proteins. The reaction mix contained 5 μL of 30x SYPRO^®^Orange dye (Invitrogen) and 45 μL of sample at 0.11 mg/mL in PBS pH 7.4. 10 μL of the mix was dispensed in quadruplicate into a 384-well PCR optical plate and was analyzed on a 7900HT real-time PCR System (Applied Biosystems) with a ramp rate of 0.5°C/min between 20°C and 99°C. Fluorescence changes in each well were measured, intensity increases plotted, and the inflection point of the slopes was used to calculate the thermostability (T_m_).

### RNA isolation and qRT-PCR

Total RNA was isolated from 50–100 mg of frozen organs or tumors with TRIzol reagent (Invitrogen). After treatment with RNase-free DNase (Invitrogen), RNA was reprecipitated, quantified by spectrophotometry and analyzed for RNA integrity by BioAnalyzer (Agilent). Total RNA was reverse transcribed using the Superscript First-Strand Synthesis Kit (Invitrogen) for RT-PCR under conditions described by the supplier. Quantitative RT-PCR was performed with inventoried mouse or human *tem1* probe (Applied Biosystems) with *18S* probe as endogenous control. The expression of *tem1* in normal organs was normalized using mouse liver or cervix as a calibrator. MS1, TC1 or 2H11 cell lysates were harvested and total mRNA extracted using Trizol, followed by DNaseI treatment and cDNA generation. Relative gene expression was calculated by the comparative CT method (2^−ΔΔCT^ method). Error bars denote standard deviation (*n*=3) or as indicated.

### Conjugation of 78Fc with Vivotag750

Conjugation of 78Fc or control human IgG (Genscript) with VivoTag-S 750 and VivoTag-S 750MAL (Perkin Elmer) was performed according to the manufacturer's instructions. Briefly, 1 mL of a 1 mg/mL antibody solution in conjugation buffer (50mM carbonate/bicarbonate buffer, pH8.5) was mixed with 30μL of VivoTag-S 750 at a concentration of 10mg/mL in Dimethylsulfoxide (Sigma) and then incubated at room temperature for 1 h. After dialysis in 1×PBS, the reaction mixture was filtered through a 0.2 μm syringe filter. Aliquots of labeled protein were stored at 4 °C in the dark until ready to analyze.

### Murine TEM1-expressing models of TC1

Subcutaneous (s.c.) tumors with upregulated expression of endogenous murine TEM1 were established by s.c. injection of 4×10^4^ TC1 cells into the right flank of nude mice. All animal experiments were conducted in compliance with Institutional Animal Care and Use Committee guidelines. Female athymic *nu/nu* mice (strain 088 Nude^−/−^, 18 g, 4-6 weeks, Charles River Laboratories) were acclimatized at the Small Animal Imaging Facility (SAIF) vivarium for 1 week before tumors were implanted. Mice were provided with food and water *ad libitum*. These TC1 cells express firefly luciferase, enabling BLI imaging. Following resections at indicated time points, excised organs and tumors were analyzed for muTEM1 mRNA by quantitative RT-PCR. These mice will develop TC1-induced s.c. tumors of approximately 5×5mm within 10 days of cell inoculation and succumb in 5-6 weeks. At day 10 after cell inoculation, 78Fc750 tracer (5 mg/kg) was injected intravenously (n=5) for longitudinal 2D planar NIR and BLI optical imaging.

Lung metastasis tumors were established by intravenous injection of 1×10^5^ luciferase expressing TC1 cells. These mice will grow BLI visible lung tumors within 7 days after cell inoculation and succumb in 3-4 weeks. Following resections at 2 wks post injection, excised lung and control organs were analyzed for muTEM1 mRNA by quantitative RT-PCR. At day 14, 78Fc750 tracer (5 mg/kg) was injected intravenously (n=5) for longitudinal 2D planar NIR and BLI optical imaging.

### Human TEM1-expressing tumor vascular model

Subcutaneous (s.c.) tumor vascular grafts expressing hTEM1 were established by s.c. injection of 2 × 10^7^ MS1-TEM1 or MS1 in each flank (for 2D study) or on the back for 3D tomography studies (upper: MS1; lower MS1-TEM1). Full details of the model has been described previously [36]. At day 14, 78Fc750 tracer (5 mg/kg) or control IgG-750 were injected intravenously for longitudinal 2D planar NIR and BLI optical imaging (n=3 per group). Mice bearing MS1-TEM1 and MS1 tumors on the back were suitable for studies using transillumination light sources and could therefore be used with dual modality tomography (DLIT and FLIT) (n=5).

### *In vivo* 2D planar Bioluminescence Imaging (BLI) and Diffuse Light Imaging Tomography (DLIT)

Mice bearing engrafts expressing fLuc reporter (MS1 or TC1 cells) were anesthetized with 1-2% isoflurane/O_2_ and BLI was performed using the IVIS Spectrum small animal imaging system (Caliper). Briefly, D-Luciferin (150 mg/mL, Gold Biotechnology) was injected intraperitoneally and optical images were acquired 15 min post-injection. Images were quantified with Living Image Version 3.0 software (Caliper). Quantitative bioluminescence imaging was performed as described elsewhere [78]. The mice bearing MS1-huTEM1 and MS1 tumors on the back underwent 3D DLIT at day 14 post engraftment (n =3). A structured light image was acquired and followed by a sequential series of images using 20 nm–wide filters centered on wavelengths from 560 to 660 nm. Three-dimensional DLIT takes into account the source emission spectrum as well as the scattering and absorption of light in tissues and can thereby calculate the 3D location and brightness of the luminescent source [79].

### *In vivo* 2D planar NIR fluorescent Imaging and Fluorescence Imaging Tomography (FLIT)

Mice engrafted with MS1 or TC1 were also imaged with the Pearl Impulse small animal imager (LI-COR), an NIR imaging system designed for use with 700 nm and 800 nm wavelengths. This system was used to measure the 78Fc750 signal at 800 nm in the longitudinal study after antibody–dye conjugate injections (1 min up to 10 days post injection). Post mortem images were taken to visualize the organ distribution of the labeled antibody.

In addition, the mice bearing MS1-huTEM1 and MS1 tumors on the back underwent FLIT to determine geometry and to quantify the depth and intensity of fluorescent sources in 3D space (n=5). First we obtained a structured light image to reconstruct the surface topography and then fluorescent images (Ex: 745nm; Em: 800nm) were obtained using multiple transillumination points and the same excitation and emission for tomographic mapping of source locations. Voxels were chosen for visualization. A generic mouse organ atlas was used to overlay the surface/source reconstruction, which allows the surface shape of the liver to be displayed. The liver voxels were later confirmed by post mortem 2D planar NIR of organs.

### *In vivo* Dual Modality Tomography (DLIT with FLIT)

The tomography source reconstruction and analyses were performed strictly according to the manufacturer's instruction (Perkin Elmer). The mice bearing MS1-huTEM1 and MS1 tumors on the back underwent, sequentially, FLIT followed by DLIT while maintaining their physical location/position on the imaging platform. The identical surface topographies from DLIT and FLIT enabled us to combine voxels from FLIT or DLIT for reconstructions. Such operations make it possible to directly visualize *in vivo* the co-localization of tumor cells (DLIT) with the 78Fc750 tracer (FLIT). Following resections, excised organs were placed in tissue cassettes and imaged under 2D planar NIR.

### Statistical analysis

Unpaired student's t-test or two-way ANOVA statistical analyses were carried out using the SPSS and StatView software packages. A value of p<0.05 was considered statistically significant.

## SUPPLEMENTARY FIGURES AND VIDEOS




