# Supplementary material for: Development, optimization, and validation of novel anti-TEM1/CD248 affinity agent for optical imaging in cancer
Source: Oncotarget. 2014 Jul 8;5(16):6994–7012. doi: 10.18632/oncotarget.2188 (PMC4196179; doi:10.18632/oncotarget.2188)
Supplement: Supplementary file 1 [file oncotarget-05-6994-s001.docx]

Development, optimization, and validation of novel anti-TEM1/CD248 affinity agent for optical imaging in cancer

**Sup Fig. 1: Conceptual design of the expression constructs of scFc78 fusions derivatives.**

The detailed domain arrangement of each protein variants is presented. LTR, long terminal repeat. RRE, Rev-responsible element. cPPT,central polypurine tract. P_SFFV_, spleen focus-forming virus promoter. SP, signal peptide. WPRE, Woodchuck hepatitis virus posttranscriptional element. P_Ubi_, ubiquitin promoter. Eme, Emerald GFP.

 **Sup Fig.2: Biodistribution profile of 125I-78Fc in tumor bearing mice.**

Mice were injected with ID8 tumor cells admixed with MS1-fLuc endothelial cells expressing huTEM1 or control MS1-fLuc cells on each flank. Two weeks later 125I-78Fc were injected intravenously as described in Figure 5C and sacrificed at the indicated time points (n=5). A, %ID/g . B, %ID/thyroid. C, fold increase of 125I-78Fc localization, shown as TEM1+ tumor vs. TEM1-negative control. Red line indicated baseline of “1”, or theoretical equal distribution among TEM1+ and TEM1- tumors.

**Excised**

**organs**

**All**

**organs**

**exposed**

**Liver/**

**kidneys**

**removed**

**Excised**

**heart/lung**

**Live**

**animal**


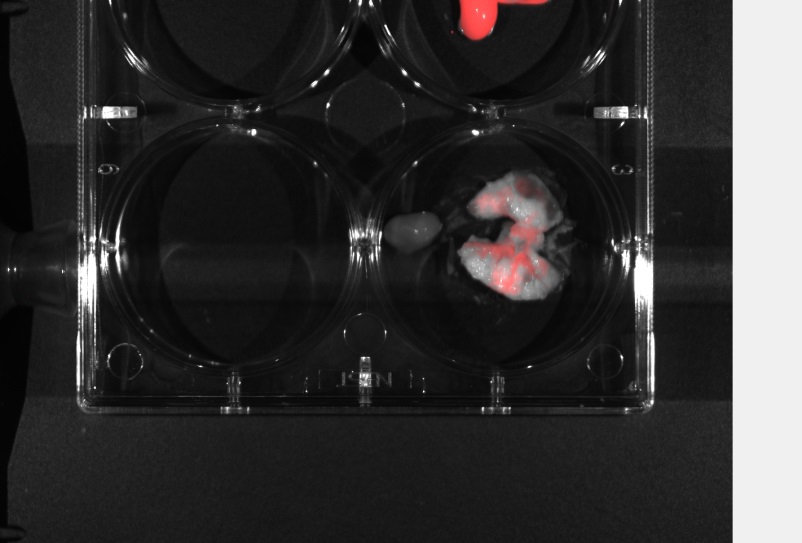

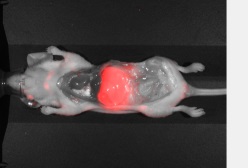

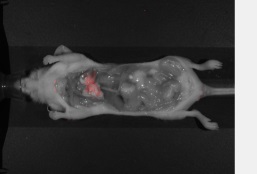

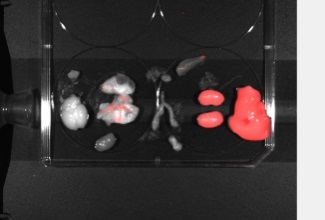

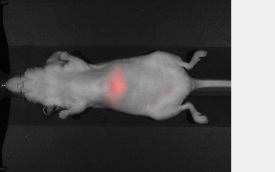


**2**

**11**

**1**

**4**

**9**

**10**

**7**

**5**

**6**

**3**

**1**

**4**

**8**

**Sup Fig 3: Whole body and organ-level NIR imaging of 78Fc -750 in mouse TC1 lung model.**

From L to R: ventral view of whole-body NIR imaging of a mouse at day 2 post 78Fc750 injection; ventral view of whole-body NIR imaging of a mouse at day 3 post 78Fc750 injection, with all organs exposed; same animal with liver and kidneys removed to visualize the signal in lung; organ-level NIR imaging; closed-up NIR imaging of heart and lung. 1. heart; 2.brain; 3. liver; 4. lung; 5. spleen; 6. kidney; 7. small intestine; 8. bladder; 9. ovary; 10. uterus; 11. thyroid; 12. TC1 sc. xenograft.


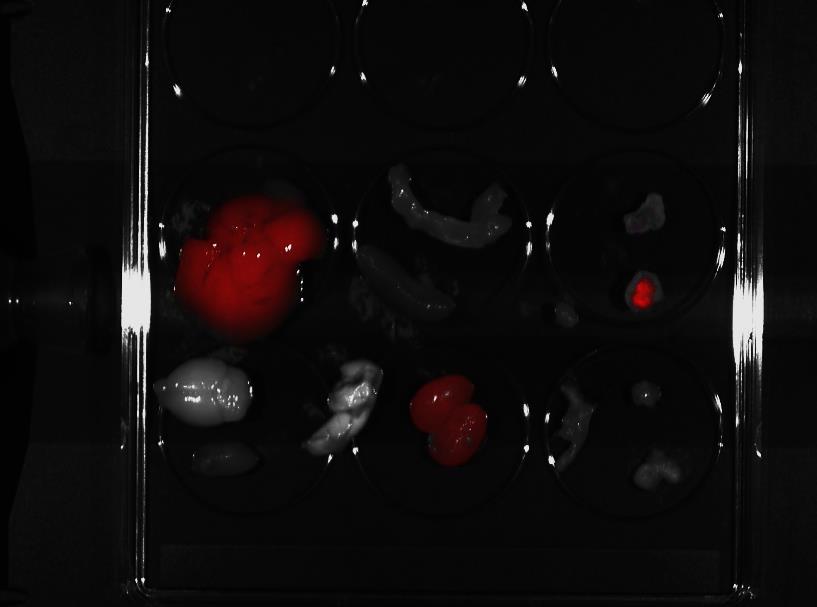


**B/W, d10 p.i.**

**B/W overlay with NIR**


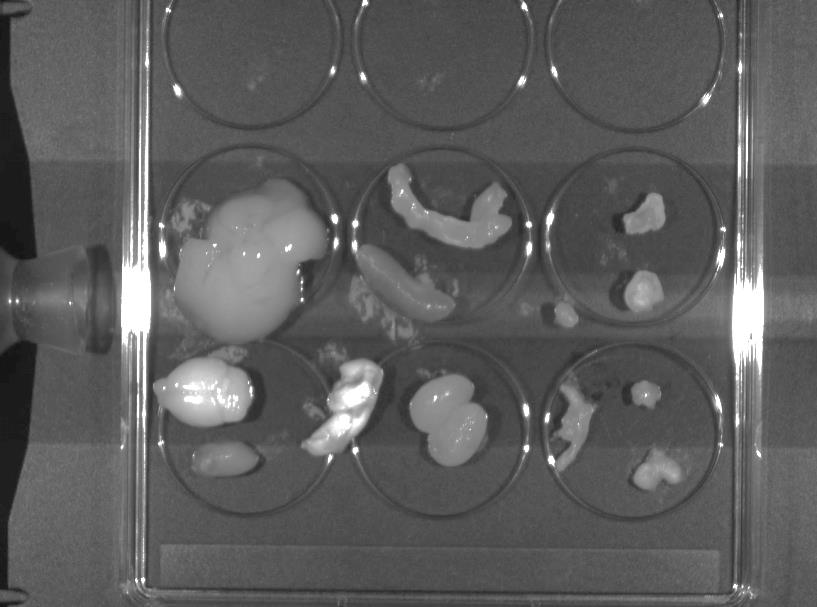


**1**

**2**

**3**

**4**

**5**

**6**

**7**

**8**

**9**

**10**

**11**

**12**

**13**

**1**

**2**

**3**

**4**

**5**

**6**

**7**

**8**

**9**

**10**

**11**

**12**

**13**

**Sup Fig 4: 78Fc NIR distribution study in mice model expressing human TEM1.** MS1-huTEM1 or control MS1 cells were injected subcutaneously into nude mice on the left or right flanks, respectively (n=3). 2-3 weeks later the mice were injected intravenously with78Fc-750 for longitudinal live animal NIR imaging study. At day 10 post injection, animals were euthanized, and organs were excised for NIR imagining. 1. heart; 2.brain; 3. liver; 4. lung; 5. spleen; 6. kidney; 7. small intestine; 8. bladder; 9. ovary; 10. uterus; 11. thyroid; 12. TEM1+ tumor; 13. control TEM1- tumor.
